# Chagas Disease and Healthcare Rights in the Bolivian Immigrant Community of São Paulo, Brazil

**DOI:** 10.3390/tropicalmed5020062

**Published:** 2020-04-17

**Authors:** Fernando Mussa Abujamra Aith, Colin Forsyth, Maria Aparecida Shikanai-Yasuda

**Affiliations:** 1 Department of Preventive Medicine, Faculdade de Medicina, University of São Paulo, Av. Dr. Arnaldo, 455. Cerqueira César; São Paulo, SP 01246-903, Brazil; 2 Drugs for Neglected Diseases initiative, Rua São José 70 #601, Centro. Rio de Janeiro, RJ 20010-020, Brazil; cforsyth@dndi.org; 3 Department of Infectious Diseases, Faculdade de Medicina, University of São Paulo, Av. Dr. Arnaldo, 455. Cerqueira César; São Paulo, SP 01246-903, Brazil

**Keywords:** Chagas disease, neglected tropical diseases, migration, healthcare rights

## Abstract

Chagas disease (CD) poses a major public health challenge for the Americas and non endemic regions around the world. This study discusses the legal framework surrounding access to healthcare for CD for Bolivian migrants living in São Paulo, Brazil. While recent guidelines stipulating care for CD exist, there is a lack of legal provisions to ensure they are regularly implemented. Bolivian migrants in SP have specific needs, including language differences and a high level of mobility. Interviews were conducted with ten participants representing public health institutions or organizations working with the Bolivian migrant community. Additionally, a review was conducted of legal, official, and health policy documents pertaining to rights of Bolivian migrants in SP. Although the right to healthcare is constitutionally guaranteed for all, in practice, immigrants, especially those without documentation, encounter barriers to initiating treatment for CD. Providing the primary health care system (SUS) card would not only improve access to healthcare for Bolivian migrants, but also provide a potential pathway toward regularization of status. The approval of clinical protocols and therapeutic guidelines for CD (2018) represents an opportunity to improve care for all Brazilians with CD. Programs with multidisciplinary teams should be developed taking into account the specific social and cultural needs of this population.

## 1. Background

Over six million people are infected with *Trypanosoma cruzi*, the parasite that causes Chagas disease (CD) [1,2,3]. Like other neglected tropical diseases (NTDs), CD is concentrated among poor, marginalized populations with limited access to healthcare. Moreover, there are substantial gaps in both research and development and public health infrastructure; effective new drugs for CD have not been developed in nearly 50 years, and less than one percent of people with the infection have received diagnosis and treatment in the Americas [4,5,6]. This leads to a considerable burden of morbidity and mortality, since, in the absence of timely identification and treatment, CD leads to serious, life-threatening complications (most often heart disease) in 30%–40% of those infected with *T. cruzi*. These complications most frequently manifest in individuals who are at the peak of their productive years (30–50 years old), creating immense voids in families and communities [7,8]. Brazil, where CD was originally discovered by Carlos Chagas in 1909, has the second-largest burden of the disease worldwide, with over 1.1 million people infected, and incurs the highest annual healthcare costs from CD, estimated at $129 million in 2013 (equivalent to $143 million in 2020) [1,9].

CD’s epidemiological profile has transformed in recent decades [10]. In the twentieth century, CD was principally confined to rural areas of Latin America. However, at the millennium’s close, political violence and economic necessity fueled rural–urban migration within Latin America as well as international migration to Europe, North America, and Asia [10,11]. Migrants in these new settings face significant political, economic, and sociocultural challenges to accessing healthcare for CD [12,13,14,15].

São Paulo, the world’s fifth largest city [16] and a major destination for immigrants from across the globe, illustrates the changing epidemiology of CD. Annual immigration to Brazil increased from 143,600 in 2000 to 268,400 a decade later [16]. Bolivia stands out as the fifth country with the highest flow of immigration to Brazil, and the city of São Paulo (SP) is the most common destination for Bolivian migrants. In effect, although the presence of Bolivian immigrants in the border areas of Mato Grosso do Sul, Mato Grosso, Rondônia, and Acre is significant, there is a high concentration of Bolivian immigrants in the metropolitan region of São Paulo.

Estimates indicate there are over 350,000 Bolivians living in SP [17]; 44.1% work in the garment industry, while others work as street vendors, doctors, and dentists [16,18]. The majority of Bolivian immigrants in São Paulo are men, who have been living in the country for more than 10 years. When arriving to São Paulo, Bolivian migrants confront a new reality of life, full of cultural, linguistic, and social differences. This situation places them in a condition of physical, psychological, and social vulnerability, which can directly influence their health status [16,19]. This vulnerability is further compounded for people who lack required documentation [20].

Bolivia, where over 600,000 people are infected with *T. cruzi*, has the highest global CD prevalence (6.1%) [1]. CD is also a major concern for Bolivian migrants living in SP. A recent study found a seroprevalence of 4.4% in this population [21]. Other major global cities where migrants face healthcare access challenges for CD include Los Angeles [22], Munich [23], Rome [24], London [25], Buenos Aires [26], and Barcelona [27].

Brazil has a long history of public health efforts devoted to CD, and domiciliary vector transmission in SP state was interrupted in the 1960s. Nevertheless, access to etiological treatment for CD via the primary healthcare system (Unified Health System, Sistema Único de Saúde, or SUS) remains a significant challenge, not only for large numbers of Brazilians who have migrated to SP from endemic areas, but for Bolivian migrants, who present unique social, political, legal, and cultural needs, and represent a new reality for SP’s healthcare system in terms of demand for health services, morbidity, and vulnerability.

The World Health Organization has made the right to health a fundamental pillar of its global strategy to combat neglected tropical diseases, while universal health coverage is considered essential to achieving the United Nations Sustainable Development Goals [28]. Nonetheless, gains in extending health coverage to vulnerable groups, especially immigrants, are in jeopardy from the rise of far-right, authoritarian-leaning governments across the globe, a phenomenon that has recently impacted Brazil. This article identifies the extent of migrants’ right to health in Brazil, and analyzes how this impacts utilization of the SUS and other public services for CD management. This work aims at improving understanding of migrants’ therapeutic itinerary for CD. The research was conducted as part of a larger study on the socioepidemiological and clinical characteristics of CD and access to healthcare among Bolivian migrants in SP [21,29,30].

## 2. Methods

Two types of primary data were used for this study: interviews of key respondents and relevant legal, technical, and policy documents.

### 2.1. Participants

Ten semistructured interviews (see semistructured interview script in the supplementary file) were conducted with respondents from the following institutions: The Secretaries of Health of the State and Municipality of SP; Bolivia’s Consul in SP; the Federal Prosecutor and Public Defender; Pastoral Migrants Service in SP, the Association of Bolivian Residents (ADRB), and the Kantuta Bolivian Association.

### 2.2. Data Collection

Interviews focused on Bolivian migrants’ health rights were conducted in 2014 and 2015 by a researcher with expertise on human rights and public health issues. The interviews followed a semistructured guide (annex) with 10 questions about respondents’ activities, particularly regarding migrants, and opinions/perspectives regarding the Bolivian community in São Paulo. Participants were selected based on their professional background and strategic position in institutions with public or official activities and programs related to Bolivian migration and the health system. Content analysis of the interviews suggested that the method reached saturation once data collection began to be repetitive. The interviews were recorded after obtaining consent from participants. Interviews were transcribed as Word documents, then analyzed and coded for themes related to interviewees’ activities, opinions, and perspectives related to the health care for migrants in SP, as well as suggestions of key documentation and references.

In addition to the interviews, we conducted a review of official legal and technical documents. From March 2014 to March 2016, we reviewed legislative and normative guidelines pertaining to the legal status of Bolivian immigrants in Brazil, as well as their health rights and possibilities to freely access Brazilian public health services, especially as pertains to CD. The review included only official government websites and legislative data from the Federal Government (www.planalto.gov.br), the Ministry of Health (http://portal2.saude.gov.br/saudelegis and http://portalms.saude.gov.br/protocolos-e-diretrizes), the Health Secretariat of the State of SP (www.saude.sp.gov.br), and the Health Secretariat of the Municipality of SP (MSP) (http://www.prefeitura.sp.gov.br/cidade/secretarias/saude/). Special attention was given to the current legal and normative technical documents that organize and structure the Brazilian public health system at the national, regional, and local levels, and to specific public policies regarding immigrants and CD.

For the documents search, key words were immigrants, immigration, Chagas disease, Clinical Protocols, Clinical Guidelines, right to health, and health rights. Our search resulted in 141 records; 62 legal and normative documents related to immigrants and 79 clinical protocols and guidelines approved by the Ministry of Health. We reviewed each record and only retained legal and normative documents related to immigration status, public policies on CD, or health surveillance (*n* = 12).

The Research Ethics Committee of the Clinical Hospital of the School of Medicine, University of SP approved the research, and the interviewees signed the respective Terms of Free and Informed Consent.

## 3. Results

The healthcare rights of Bolivians with CD living in SP depend on three important frameworks: (1) their legal status in Brazil, particularly as it pertains to accessing healthcare via the SUS; (2) policies and legal requirements regarding provision of testing and treatment for CD; and (3) capacity of the health system to provide care for Bolivians with CD.

### 3.1. Legal Status Considerations for Bolivian Migrants in SP

The legal and normative documents related to immigrants, collected during the research provided insight on how Brazilian legislation treats Bolivian immigrants regarding the right to health.

Brazilian Law 6.815/1980 (known as the Foreigners’ Statute) [31] indicates visas may be granted to foreign nationals who enter Brazil and meet certain legal requirements. Article Five of the Federal Constitution affirms that foreign nationals living in Brazil enjoy the same legal status and rights as Brazilians. However, whenever required by any authority or agent, foreigners must show official documentation attesting to their legal permission to reside in Brazil. In cases of irregular entry or unauthorized residence of a foreign national in Brazilian territory, authorities will first demand voluntary removal, and failing this, will initiate deportation procedures.

Obtaining the necessary documents to remain in Brazil poses a significant challenge, putting at risk migrants’ right to access healthcare. First, to obtain official residence in Brazil, a migrant must first produce original personal documents from their country of origin. These documents can sometimes only be obtained through the Bolivian Consulate. After that, a series of bureaucratic processes including forms and payments must be completed through the Brazilian Federal Police Department. This includes going in person to the local police office. These cumbersome and potentially intimidating processes can make it very difficult for migrants to regularize their status. According to one respondent from an NGO working with Bolivian migrants, “there are some bureaucratic obstacles in the Brazilian and Bolivian governments that make it difficult to obtain documents, and this ends up having repercussions on the enjoyment of rights”.

Brazil’s Migration Amnesty Law [32] provides an opportunity for undocumented migrants who had entered the country prior to February 1, 2009, to regularize their status and obtain freedom of movement, the right to work, and access to public services, including healthcare. While this represented a potentially strong advance in the protection of the rights of Bolivian and other immigrants, interviewees report that a lack of documentation continues to be a major concern. The Bolivian General Consul estimates there are around 50,000 Bolivians in SP in need of regularizing their status, and not having proper documentation represents a major barrier to accessing public health services. Interviewees indicated Bolivian immigrants often feel strong apprehension or fear that attempts to access healthcare will expose their status, subjecting them to repercussions including fines and deportation. As reported by one Bolivian migrant representative, “if I give my address and this kind of information in the hospital, not being legal in the country, they will go after me as soon as they realize I do not have the proper visa to be in Brazil and they will want to send me back”. One of the public officials interviewed confirmed this reality: “Migrants without proper documents can legally be submitted to some enforcement measures such as arrest or deportation”.

Thus, although immigrants’ access to healthcare is guaranteed as a constitutional right, this is in large part negated by the difficulty of obtaining the documentation needed to secure legal residency. As reported by a public official responsible for immigration issues, “sometimes the migrant comes to us to ask for a kind of document assuring that they have the right to be treated in SUS facilities even if they do not have legal status in the Country …; and they come to us because they have already tried to be treated and the public service denied access because they do not have the proper documents to show”. It is important to state that migrants in Brazil, even undocumented, have the constitutional right to access public health services (even if they do not show any identity document and proof of residence). In these cases, they can go to public defenders or to the Public Ministry to ask for a document able to protect their right to health. Although this documentation can improve access from a legal standpoint, other linguistic, sociocultural, and CD-specific healthcare barriers, remain.

### 3.2. Legal Status and Healthcare Access

According to articles 5 and 196 of the Brazilian Constitution, the access of Bolivian migrants to public health services must occur in the same way as for Brazilian citizens. Migrants in Brazil, regardless of status, have the same health rights as Brazilians, including access to health services and treatments. Table 1 outlines the major portals of entry by which Brazilians or Bolivians living in SP might enter the SUS [33] as defined by governmental laws, which standardize the entry process. Whenever a new patient enters through one of these doors, they will receive a SUS card, entitling the recipient to: primary care services from Family Health Strategy facilities; office visits for primary care services, including vaccinations; home visits conducted by the Family Health Strategy; emergency care; and mental health services [34].

The Family Health Strategy (FHS) seeks to promote the quality of life of the Brazilian population and address factors that put health at risk, such as lack of physical activity, poor diet, and the use of tobacco. With comprehensive, equitable, and continuous care, the FHS serves as a gateway to the public Unified Health System (SUS) in Brazil. The FHS is composed of a multidisciplinary team that has at least one general practitioner or family health doctor, one nursing assistant, two nursing technicians and several community health agents (CHAs) to cover 100% of the registered population with a maximum of 750 people per agent and 12 CHAs per Family Health team [34]. There can also be an oral health team, consisting of a general dentist and a technician in oral health. Each Family Health team is responsible for a maximum of 4000 people from a given area [34].

Many facilities within the health system will provide the card needed to utilize SUS healthcare services regardless of documentation status. In fact, all interviewees reported the SUS card was often the first official Brazilian document obtained by Bolivian migrants from a public authority, emphasizing that access to the health system may represent the first contact with the State and a potential starting point for status regularization. Within the SUS, there is no regulation stipulating that foreigners receive different types of care than Brazilian nationals. Municipal and state health authorities in SP also indicate that there is no specific therapeutic protocol pertaining to care for immigrants.

### 3.3. Legal and Policy Framework for CD Healthcare

In 2011, Brazilian Law 12.401 mandated the creation of official clinical protocols and therapeutic guidelines for specific diseases [33]. While Brazilian experts had published two consensus documents on CD [8], the Ministry of Health had not put forth a clinical protocol for CD during the period of data collection. Aside from the consensus documents, there were basic manuals of care for CD [35], and regional guidelines for care of CD cardiomyopathy [36] and oral CD [37], but there were no legal provisions to enforce the recommendations in these documents. However, on 30 October, 2018, a clinical protocol for diagnosis and therapeutic guidelines (Protocolo Clinico de Diretrizes Terapeuticas, PCDT) for CD was approved by the National Commission for Incorporation of Technology into the SUS (Comissão Nacional de Incorporação de Tecnologias no SUS, or CONITEC) [38]. The PCDT contained several provisions to strengthen access to healthcare for people with CD, including obligatory reporting of chronic cases, measures to reinforce referral pathways and provision of etiological treatment at the primary level, and strengthening of recommendations to etiologically treat chronically infected adults up to age 50, especially women of childbearing age. The PCDT does not contain special provisions for migrants in the main document, so its recommendations are valid for all individuals registered in the SUS. However, the tone of the PCDT’s supporting annexes suggests that routine treatment would not be recommended for migrants, based on a lower rate of PCR conversion after antiparasitic treatment in Argentina, Bolivia, and Colombia compared to Brazil [39]. As this data is based on the analysis of only one study population, whereas other observational studies in different contexts do suggest clinical benefits from antiparasitic treatment in various populations [40,41,42,43,44], it deserves further discussion, lest this provision impede the ability of migrants to enjoy the right to healthcare in the form of antiparasitic treatment.

### 3.4. Needs within the Health System

Interviews revealed several challenges within the health system regarding provision of care for CD to Bolivian migrants. Table 2 displays the challenges highlighted by the interviewees, identifying the major gaps and related outcomes.

## 4. Discussion

Requiring documentation poses a major deterrent for Bolivian immigrants who do not enjoy regular status, as Brazilian health facilities require one document of identification and one proof of residence in the city to proceed with treatment. This protocol can be more flexible when the Family Health Team visits the patient, since in these cases proof of residence is not required. Additionally, the different epidemiological profile of the disease in Bolivia could create different clinical issues; for example, women who have resided in endemic areas may have a greater risk of transmitting CD to their infants in utero. In fact, prior research found a 6.1% prevalence of *T. cruzi* infection in Bolivian women of reproductive age living in São Paulo [21], substantially higher than reported prevalence among Brazilian women in endemic areas (1.1%) [45]. Moreover, some clinical parameters including morbidity may differ between people infected in Bolivia or Brazil, as reported [29], largely due to the younger age of the Bolivian migrant population with CD. Interviewees also stressed provider–patient language differences as an important barrier, rendering it difficult for patients to understand explanations of their diagnosis or instructions for treatment. In addition, like other migrant populations, Bolivians with CD are highly mobile, which can make long-term follow-up challenging. Mobility from São Paulo to Bolivia or from one district to another in the city of São Paulo has contributed to a high rate (42.1%) of abandonment of antiparasitic treatment [29].

One of the main gaps revealed in our research was the lack of a specific therapeutic protocol (PCDT) for CD, despite the stipulation in favor of creating such a protocol by Brazilian Law 12.401 in 2011. The approval of the PCDT for CD in October, 2018 [38], presents an opportunity to increase access to care for CD for all affected people in Brazil. However, neither the PCDT, nor the prior legal framework, contain special provisions relating to the care of migrants. Respondents indicated that there are no provisions or programs in place to ensure migrants from known endemic areas receive timely diagnosis and care.

As the results illustrate, another important gap is related to the right of immigrants to obtain healthcare on an equal footing with Brazilian citizens, as affirmed by Brazil’s Constitution. In practice the requirement for producing documentation in order to receive services poses a key barrier, even considering that this is not a uniform practice. Some providers related to Family Health Teams do not require documentation prior to seeing patients, whereas others, related to Health Facilities, turn away undocumented patients.

In different countries and health systems, migrants with CD have faced key challenges to accessing care and often represent a particularly vulnerable group. In the United States and Europe, for example, immigration status can pose a major barrier to obtaining care for people with CD [12,14,46,47]. In Brazil, bureaucratic processes to obtain legal documents are potentially intimidating and make it very difficult for migrants to regularize their status.

Moreover, CD has persistently remained a neglected disease despite the existence of universal health coverage in endemic [6] as well as nonendemic countries [46]. In addition to legal barrier and gaps in healthcare systems, migrants with CD must navigate linguistic and cultural differences with healthcare personnel. People diagnosed with CD may experience fear regarding the implications of the disease and stigma because of its association with poverty and rurality [13].

It is worth highlighting that the health system of the MSP has the capacity to treat CD in both immigrants and Brazilians. There is, therefore, the practical possibility of creating well-defined therapeutic itineraries for CD in SP and, thereby reducing the risks of aggravation of this disease in diagnosed patients. We propose positioning the public health system as a port of entry to guarantee immigrants’ right to health, and other rights, through the Family Health Strategy [34]. Through this channel, the SUS card could be provided, not only assuring better access to health services but, in many cases, also providing the first official document in Brazil, facilitating a pathway toward regularization of status. Ideally, coverage through the Family Health Strategy in the city of São Paulo will allow teams to identify the immigrant population and their health needs, strengthening the health relationship with this group.

Significant improvements in access to treatment for CD for the Bolivian community will depend on collaboration and dialogue between health services; municipal, state, and federal authorities; the Bolivian Consulate, and organizations working within SP’s Bolivian community. Special programs that take into account the sociocultural needs of the Bolivian community are needed, and such programs should include training of local healthcare personnel to improve interactions with Bolivian patients.

In the interest of promoting an open society supporting those seeking better lives and working conditions, the Federal Constitution assures immigrants the same rights as Brazilian citizens, and also guarantees healthcare as a human right [48]. The management/treatment of Bolivian immigrants by the SUS is therefore a duty of the state and society. A legal framework exists supporting the right of Bolivian migrants to obtain healthcare for CD, which could potentially produce an important reduction in morbimortality and an improvement in quality of life. However, important challenges remain before Brazil’s immigrant community can freely exercise their right to treatment for CD and other diseases.

Finally, a key limitation of this work is the low number of interviewees, although data analyses suggested that the saturation level was reached since some information became repetitive. This is possibly explained because the invited responders are in strategic positions concerning health care access in the city of São Paulo. Nonetheless, further ethnographic research with people affected by CD in São Paulo, as well as workers within the health system, could illuminate other important aspects of migrants` access to health.

## 5. Declarations

**Ethical Approval:** The Research Ethics Committee of the Clinical Hospital of the School of Medicine, University of São Paulo approved the research (N°. 196.698/2013).

**Consent for Publication:** Not applicable.

**Availability of Data and Materials:** The datasets used and/or analyzed during the current study are available from the corresponding author on reasonable request.

## Figures and Tables

**Table 1:** Entry points to the Unified Health System (Sistema Único de Saúd—SUS) for Bolivian immigrants in the city of São Paulo, Brazil.

SUS Entry Points	Health Services
Primary Healthcare Facilities	• Outpatient care • Laboratory tests • Pharmacies • Vaccination programs • Other basic health services
Family Health Strategy (FHS)	• Home visits by FHS teams • In-person care at SUS clinics generated by FHS home visits
Emergency Units	• Emergency services (emergency room)
Center for Psychosocial Care	• Mental health services

**Table 2:** Gaps in care of Bolivian immigrants with Chagas disease in São Paulo’s public health system.

GAPS	Description in the Interviews	Outcomes
1. Requiring documentation discourages patients from utilizing primary healthcare services.	“We know of those who are registered, but we know that there are many who do not have documents” (Interviewee* 1). “In relation to foreigners, we have a problem with the SUS card. Many foreigners have difficulty understanding the SUS system, understanding how they have access.” (Interviewee* 2).	Missed opportunity to provide timely treatment and reduce morbidity/mortality. Missed opportunity to halt vertical transmission Non-attendance.
2. Unitary approach to healthcare for Chagas disease.	“There is a large concentration of migrants, but they do not go to the doctor. Because they are mistreated, they do not go (…)” (Interviewee* 3).	Failure to address different needs in terms of morbidity according to the country of origin.
3. Language differences. Lack of professionals able to communicate in languages other than Portuguese.	“The main one would be regarding language, healthcare services in Brazil are poorly paid, people are stressed, they are poorly paid, someone who does not know the language arrives and is already mistreated” (Interviewee* 3). “Firstly, we identify where our Bolivian patient is located with our teams, so we have already seen that there is a problem with the language filling in the forms, so the materials produced are now also being made in Spanish.” (Interviewee* 4). “They complain a lot about prejudice, but I think that the biggest problem is that of language, which generates ill will among public workers who serve them”. (Interviewee* 5).	Non-comprehension of the diagnosis or the therapy to be followed. Lower intention to search for the health service. Decreased adherence to the treatment.
4. Movement of immigrants within and beyond the city.	“Immigrants are spread over the Administrative Districts: Brás, Sé, Vila Maria and Vila Guilherme, mainly” (Interviewee* 6). “First, we know that they are located in the last two decades, in the region of the East Zone, Center, more towards the East Zone, Mooca, Brás, go to Penha, Guarulhos, there is a concentration there. (…). And also, without a doubt, we have those inside. In Carapicuíba we also have, a little in Osasco, we even have Americana already. And Guarulhos without a doubt, and all continuity in São Paulo. Penha, from where Guarulhos continues, we have a large concentration of Bolivians who are working here on sewing” (Interviewee* 7).	Incorrect or outdated addresses and contact information. Greater challenges adhering to long-term treatment regimens.
5. Lack of reference and counter-reference health services for patients diagnosed with CD.	“Routine examinations are not yet carried out on Chagas disease, but there is already a discussion on the problem and guidance to obstetricians and gynecologists to request the examination in adults with epidemiological history and pregnant women”. (Interviewee* 6). “To my knowledge, there is no reference and counter-reference network. A network does not exist. Some individual initiatives have…If there is a group developing some research, it opens an outpatient clinic to assist the patient. It is the units that are the school centers, right? For research.” (Interviewee* 4).	Lack of specific guidelines within the public health system for the management of CD patients. Lack of prior case histories and other clinical information for patients who are diagnosed with CD, or for previously diagnosed patients who need to see specialists due to complications.

• *Interviewee* 1: Representative of the Migrants Pastoral Service in SP; Interviewee* 2: Representative of Federal Public Defender Office; Interviewee* 3: Representative of Bolivians Association of Kantuta (São Paulo City); Interviewee* 4: Primary Care Center Director on São Paulo City, Interviewee* 5: Representative the Human Rights Committee of the São Paulo City Parliament; Interviewee* 6: Basic Care Coordinator of São Paulo City, Interviewee* 7: Representative of Bolivia`s Consulate São Paulo, Brazil.

## Supplementary Materials

The following are available online at https://www.mdpi.com/2414-6366/5/2/62/s1, Semi-structured interview script.
