# Supplementary material for: Chagas Disease and Healthcare Rights in the Bolivian Immigrant Community of São Paulo, Brazil
Source: Trop Med Infect Dis. 2020 Apr 17;5(2):62. doi: 10.3390/tropicalmed5020062 (PMC7345312; doi:10.3390/tropicalmed5020062)
Supplement: Supplementary file 1 [file tropicalmed-05-00062-s001.pdf]

Supplementary Materials

Chagas Disease and Healthcare Rights in the Bolivian Immigrant Community of São Paulo, Brazil

Semi-structured interview script:

1. What is your knowledge about the Bolivian population in the Municipality of São Paulo?

    1.1. Please, specify with regard to:

    a. The location of Bolivian migrants in the municipality.
    b. Number of Bolivian migrants.
    c. Main health demands.

2. Do you know demands related to Chagas disease? If so, comment Institutional or informal ways to protect the health of migrants.

3. In what way you or your institution act with regard to the health demands of Bolivian migrants in the municipality of São Paulo?

4. How do you refer or would refer a demand related to the treatment of Chagas disease?

5. What are the strategies of action in the defense of the rights related to the health of Bolivian migrants that you believes to be the most effective.

6. What are the strategies of action in defense of the rights related to the health of Bolivian migrants that you believes to be the least effective.

7. What public health policies do you have? Believes necessary to improve the access of Bolivian migrants to the health system in the municipality of São Paulo.
